
JOURNAL INFORMATION
==============================
NLM Title Abbreviation: Innov Surg Sci
Journal ID: Innov Surg Sci
NLM Journal ID: 101708165
Journal ID: iss

Innovative Surgical Sciences

EISSN: 2364-7485
Publisher: De Gruyter

ARTICLE INFORMATION
==============================
PMCID: PMC11005946
DOI: 10.1515/iss-2024-2006
Article ID: iss-2024-2006
Article version: 1
Subjects: Article

Abstracts DGCH

Electronic publication date: 2024 Apr 9
Publication date: 2024 Apr
Volume: 9
Issue: Suppl1
First page: 253
Last page: 279
Copyright: © 2024 by Walter de Gruyter Berlin/Boston
Copyright year: 2024
Copyright holder: De Gruyter
License: This work is licensed under the Creative Commons Attribution 4.0 International License.
License URL: https://creativecommons.org/licenses/by/4.0/

Page count: 27

==============================

Abstracts – DCK 2024 – 141st Congress of the German Society of Surgery – Leipzig, April 24–26 • DOI 10.1515/iss-2024-2006
Innov Surg Sci 2024; 9 (Special Suppl 1): s253–s279

**Influence of geriatric multimorbidity on the clinical outcome in patients after non-operatively and surgically treated proximal humeral fracture**
ID: 306

Jan-Philipp Happe[1 2], Karen Fischhuber[2 3], Josef Stolberg-Stolberg[1 2], Janette Iking[1 2], Ursula Marschall[4], Michael Raschke[1], Jeanette Köppe[2 3], J. Christoph Katthagen[1 2]

[1]*Universitätsklinikum Münster, Klinik für Unfall-, Hand- und Wiederherstellungschirurgie, Münster, Deutschland*, [2]*Universität Münster, Arbeitsgruppe "Mathematical Surgery", Münster, Deutschland*, [3]*Universität Münster, Institut für Biometrie und Klinische Forschung, Münster, Deutschland*, [4]*Barmer, Institut für Gesundheitssystemforschung, Berlin, Deutschland*

**Background:**
In Germany, the proportion of the population with an age of  65 years and more is expected to increase from 21% to 32% by 2060. This demographic change is also expected to lead to an increase in geriatric care (1). Currently, there are different approaches defining geriatric patients. One of these approaches is the concept of geriatric multimorbidity (GtMM), which is characterised by the presence of geriatric characteristic complexes (GtMKs). An increasing proportion of people over 65 years of age is also likely to increase the number of fractures, with the proximal humeral fracture (PHF) being the third most common fracture in older people (2,3). Therefore, we aimed to evaluate,  if geriatrics-typical characteristic complexes are suitable for assessing the course of patients with PHF, who have been treated non-operatively or surgically.

**Materials and methods:**
Data of the BARMER health insurance from 01/2011 to 12/2020 were analysed retrospectively. All patients aged 65 years and older, who received non-operative or surgical treatment for a proximal humeral fracture (ICD: S42.2) were included. Within the two years prior to diagnosis, GtMKs were recorded using ICD-10 GM and OPS codes. Individuals above the age of 70 years with at least 2 out of 15 GtMKs were classified as geriatric according to the "Delineation Criteria of Geriatrics" version V1.3 (4). The endpoints were defined as overall survival (OS), major adverse events (MAE), thromboembolic events (TE) and injury/surgical complications. Event rates were determined using Kaplan-Meier estimates and cumulative incidence functions using Aalen-Johansen estimates. Multivariable analyses were performed using Cox regressions and Fine-Gray models.

**Results:**
A total of 78,172 patients were included (median age 78; 84% female), of whom 44,050 (56.4%) were treated non-operatively and 34,122 (43.6%) received surgical treatment. Patients younger than 70 years had in  median 2 GtMks  and a median of 3 GtMKs was observed in those older than70 years of age. The absolute number of GtMKs at PHF diagnosis had a dramatic impact on the further course. After adjustment for patient risk profile, the presence of 2 GtMKs was still associated with a comparable risk to GtMK-free patients in terms of OS (HR=0.99; 95% CI: 0.93-1.06; p=0.820), but then monotonically increased to a HR=2.57 (95% CI: 2.28-2.90; p<0.001) with over 9 GtMKs. In patients with surgical treatment, a lower mortality was observed (HR=0.89; 95% CI: 0.86-0.91; p<0.001). Other endpoints showed  similar trends. There were also differences between specific expressions of the GtMks. For example, "cognitive deficits" (HR 1.62; 95% CI: 1.57-1.67; p<0.001) and "malnutrition" (HR 1.50; 95% CI: 1.42-1.59; p<0.001) were associated with worse survival.

**Conclusion:**
In patients with a proximal humeral fracture, the severity and individual severity of geriatric multimorbidity was a risk factor for a fatal outcome, regardless of the treatment. However, large differences in the specific GtMKs were evident. Therefore, a holistic therapy approach is urgently recommended for geriatric patients.

Open Access. © 2024 The Author(s), published by De Gruyter. This work is licensed under the Creative Commons Attribution 4.0 International License.

**Literature:**

1. Breidenbach P, Kaeding M, Schaffner S. Population Projection for Germany 2015–2050 on Grid Level (RWI-GEO-GRID-POP-Forecast). Jahrb Für Natl Stat. 26. Juli 2019;239(4):733–45.
2. Launonen AP, Lepola V, Saranko A, Flinkkilä T, Laitinen M, Mattila VM. Epidemiology of proximal humerus fractures. Arch Osteoporos. 2015;10:209.
3. Curtis EM, Moon RJ, Harvey NC, Cooper C. The impact of fragility fracture and approaches to osteoporosis risk assessment worldwide. Bone. November 2017;104:29–38.
4. Borchelt, M. et al. Abgrenzungskriterien der Geriatrie - Version V1.3. (2004)

**The Treatment and Outcome of Centenarians with Proximal Humeral Fracture**
ID: 522

Jeanette Köppe[1], Julia Sußiek[2], Josef Stolberg-Stolberg[2], Karen Fischhuber[1], Janette Iking[2], Ursula Marschall[3], Michael Raschke[2], Jan Christoph Katthagen[2]

[1]*Institut für Biometrie und Klinische Forschung (IBKF), Universität Münster, Münster, Deutschland*, [2]*Klinik für Unfall-, Hand- und Wiederherstellungschirurgie I UKM, Universitätsklinikum Münster, Münster, Deutschland*, [3]*BARMER Institut für Gesundheitssystemforschung, Wuppertal*

**Background:**
As life expectancy increases, patients with age-related fractures are also getting older. This makes the treatment of patients with an age of 100 and more increasingly challenging. However, due to the currently small number of patients, this cohort has hardly been described in the literature so far. Therefore, our aim was to describe patients over 100 years of age with proximal humeral fracture (PHF) and to evaluate their outcome based on a large, nationwide data source.

**Materials and methods:**
Health claims data of the German BARMER Health Insurance from 01/2005 – 12/2022 were retrospective analysed. All elderly (age ≥ 65 years) patients with a coded diagnosis of PHF (ICD: S42.2) from 01/2011 – 09/2022 were included to the study. Patients with an age of 100 years and more are selected; younger patients (65 – 99 years) were used as a control group. Patients were further divided into non-operative treatment and surgical treatment using locked plate fixation, reverse total shoulder arthroplasty or other surgical treatment variants. The treatment during the first 21 days after diagnosis was decisive for allocation. Patients were observed from the time of the first diagnosis of PHF to end of follow-up (defined by death from any cause, end of study (31/12/2022) or exit from the database). Overall survival (OS), major adverse events (MAEs; defined as acute myocardial infarction, stroke, resuscitation, cardiac arrest, acute renal failure, acute liver failure, acute respiratory distress syndrome, sepsis or death from any case), injured-related/surgical and minor outpatient complications were defined as primary outcomes. Cumulative incidence functions were determined using Aalen-Johansen estimates, while death was considered as a competing risk event for surgical complications. Associations between age and primary endpoints were analysed using multivariable Cox regression models. For surgical complications, Fine & Gray Cox models were used and the respective sub-distributional hazard ratios (HRs) were presented.

**Results:**
Of the 99,134 included patients, 198 were 100 years and older. The centenarians were more often female (age ≥ 100 years: 89% with female sex vs age <100 years: 84% female sex), were less often diagnosed in an outpatient setting (age ≥ 100 years: 30% vs age<100 years: 40%) and were less often treated surgically (age ≥ 100 years: 22% vs age<100 years: 46%). In general, with increasing age, the rate of patients with surgical treatment decreased. Patients with an age of 100 years or more are more often diagnosed with osteoporosis (42% vs. 33%) but received less often a pharmaceutical anti-osteoporosis therapy (8% vs 12%), especially lower prescription rates of bisphosphonates.
Even though centenarians were more affected from age-associated comorbidities – e.g. dementia (33% vs 12%), chronic heart failure (50% vs 20%) or chronic kidney disease (32% vs 22%) – life-style associated risk factors are less common in those patients with an age over 100. In detail, alcohol abuse (<1% in centenarians vs 4%), nicotine abuse (<1% in centenarians vs 6%), obesity (3% vs 19%), diabetes mellitus (25% vs 30%) and atherosclerosis (13% vs 16%) were less often coded during the evaluated pre-phase of 24 month before PHF.
Concerning the outcome during further course after fracture, overall survival was decreased with increasing age (rate of death: 65 – 69 years 2.8%, 70 – 79 years 5.0%, 80 – 89 years 12.9%, 90-99 years 29.2%, age ≥ 100 years 52.9%; p<0.001), which was also confirmed using multivariable regression analysis. Similar effects were found for major adverse events.

Moreover, with increasing age, lower rates of injured-related/surgical complications (65 – 69 years 10.9%, 70 – 79 years 10.9%, 80 – 89 years 9.3%, 90-99 years 7.3%, age ≥ 100 years 6.2%; $p<0.001$) and lower rates of minor outpatient complications (65 – 69 years 32.4%, 70 – 79 years 29.8%, 80 – 89 years 22.2%, 90-99 years 13.2%, age ≥ 100 years 9.3%; $p<0.001$) were observed. Using multivariable regression analysis to adjust for patient's individual risk profile, the observed effects were confirmed, i.e. increasing age was associated with lower injured-related/surgical and minor outpatient complications

**Conclusion:**
Treatment of PHFs in centenarians demands difficult decision-making for medical personnel. Our data shows that a relevant proportion were treated surgically. Low complications confirm surgery as a viable option. However, technical challenges such as implant fixation in poor quality bone will remain an area of difficulty in future. Furthermore, a high rate of geriatric comorbidities underlines that specialized geriatric co-treatment is indispensable for these patients. Eventually, high mortality of centenarians indicates that treatment decision remains highly individual and patient‘s treatment wish must be respected.

### The importance of ileus-prophylactic surgery for metastasized small intestinal neuroendocrine tumours

ID: 133

Lukas Kürmann[1 2], Harald Lahner[2 3], Nicole Unger[2 3], Sarah Theurer[2 4], Wolfgang Fendler[2 5], Ken Hermann[2 5], Dagmar Führer-Sakel[2 3], Henning Dralle[1 2], Ulf Neumann[1 2], Frank Weber[1 2]

[1]*Universityhospital Essen, Department of General-, Visceral- and Transplantation Surgery, Essen, Deutschland*, [2]*Universityhospital Essen, ENETS Center of Excellence, Member of EUROCAN, Endocrine Tumor Center at West German Cancer Center, Essen, Deutschland*, [3]*Universityhospital Essen, Department of Endocrinology, Diabetes and Metabolism, Essen, Deutschland*, [4]*Universityhospital Essen, Institute of Pathology and Neuropathology, Essen, Deutschland*, [5]*Universityhospital Essen, Department of Nuclear Medicine, University Hospital Essen, Essen, Deutschland*

**Background:**
Small intestinal neuroendocrine tumour (SI-NET) is a rare tumour entity and many patients present with distant metastasis at time of diagnosis. It is of debate if the resection of the primary tumour in stage IV disease offers an advantage. The objective of this study is to investigate the benefit of ileus-prophylactic surgery for SI-NET.

**Materials and methods:**
52 patients (m=22; f=30) surgically treated at our department between 2015 -2020 (5 years) were enrolled in this retrospective study. The mean follow-up time was 4.2 years.

**Results:**
The median age was 58.8 years (39 to 81 years). 66% of patients already harboured liver metastases at time of diagnosis. The indication for surgery was under curative intent for 17 patients (33%, group 1) and ileus-prophylactic for 32 patients (61%, group 2). Three patients required emergency surgery for intestinal obstruction. The median tumour size was 1.75 cm and 94% of tumours were in the distal ileum. Tumour grade was G1 for 72% and G2 for 24% of patients. The 5-year cumulative survival rate was 100% for group 1 and 94% for group 2 ($p=0.292$). 27% of all patients showed complete response and 61.5% had stable disease, while 11.5% experience progressive disease. Overall, 36% of patients developed tumour progression during follow-up. The mean progression-free survival (PFS) was 58,3 months (95% CI 48,3 – 68.3). Which was significantly better in group I (mean PFS 82months) compared to group 2 (mean PFS 44 months) ($p<0.001$). All patients that received emergency surgery experienced tumour progress with a mean PFS of 16 months after surgery. Importantly, while 15 of 32 patients (47%) of group 2 showed tumour progression only one of 17 patients (6%) in group 1 developed metastases during follow up ($p=0.013$). Progression in group 2 occurred predominantly in the liver (79%), while isolated lymphatic progression was rare (7%). Of note, 12 of 32 patients (37.5%) that received ileus-prophylactic surgery showed intraoperatively signs of intestinal obstruction or maleperfusion. This was observed in 3 of 3 patients (100%) that received emergency surgery but only 3 of 19 patients of group 1 (15.7%) ($p=0.001$). Thorough re-evaluation of patients´ charts identified signs for potential subileus in 14 of 52 patients with a significant correlation between the presence of those slight clinical signs and the intraoperative and pathological findings for intestinal obstruction ($p<0.001$).

**Conclusion:**
Our study provides further evidence in favour for surgery for SI-NET even in the setting of liver metastases. Ileus-prophylactic surgery is associated with an excellent overall survival and the radical resection of the primary tumour in combination with vessel-preserving lymphadenectomy helps to control disease outside of the liver. Furthermore, about 30% of patients already showed evidence for intestinal obstruction. Therefore, we consider ileus prophylactic surgery in SI-NET as part of the

Abstracts – DCK 2024 – 141st Congress of the German Society of Surgery – Leipzig, April 24–26 • DOI 10.1515/iss-2024-2006
Innov Surg Sci 2024; 9 (Special Suppl 1): s253–s279

therapeutic concept. As tumour progression occurs frequently, therefore close follow-up and a multi-disciplinary management is vital for these patients.

**Portalvenous Contrast enhancement ratio of the adrenal glands and spleen as prognostic marker of mortality in patients with acute mesenteric ischemia**
ID: 683

Hans-Jonas Meyer[1], Daniel Seehofer[2], Timm Denecke[1], Matthias Mehdorn[2]

[1]*Uniklinik Leipzig, Diagnostische und Interventionelle Radiologie, Leipzig, Deutschland*, [2]*Uniklinik Leipzig, Klinik und Poliklinik für Viszeral-, Transplantations-, Thorax- und Gefäßchirurgie, Leipzig, Deutschland*

**Background:**
Contrast enhancement of the adrenal gland defined by computed tomography (CT) was previously analyzed as a prognostic factor for critically ill patients due to various diseases. However, no study investigated this quantitative parameter in patients with acute mesenteric ischemia, a potentially lethal disease. Therefore, the aim of this study was to evaluate the prognostic value of the contrast enhancement of the adrenal glands in patients with clinically suspected AMI.

**Materials and methods:**
All patients with clinically suspected AMI were retrospectively assessed between 2016 to 2020. All patients underwent surgical exploration. Overall, 134 patients (52 female patients, 38.8%) with a mean age of 69.2 ± 12.4 years were included into the present analysis. For all patients, the preoperative CT was used to calculate the contrast media enhancement of the adrenal glands and the spleen.

**Results:**
Overall, 27 (20.1%) patients died within the 24-hour period and 94 (70.1%) within 30-day. There were statistically significant differences regarding the mean values for adrenal-to-spleen ratio for 24h-mortality (p=0.001) and 30-day mortality (p=0.004), whereas the radiodensity of the inferior vena cava and the radiodensity of the spleen was statistically significant between survivors and non-survivors after 30-days (p=0.037 and p=0.028, respectively). In Cox regression analysis mean adrenal radiodensity was associated with mortality after 24h with a HR of 1.09 (95% CI 1.02-1.16, p=0.01).

**Conclusion:**
The contrast media enhancement of the adrenal gland is associated with the 24h- and 30-day mortality in patients with AMI. However, the identified associations are worse compared to previous analyses regarding other critically diseases.

**Augmented Reality in Pelvic Surgery – using Microsoft's® HoloLens 2 as intraoperative radiation-free navigation tool**
ID: 179

Vincent Schenk[1 2], Christof Audretsch[2], Arlen Siegemund[2], Leyla Tümen[2], Tina Histing[2], Alexander Trulson[3], Steven Herath[2], Markus Küper[2]

[1]*Eberhard Karls Universität Tübingen, Tübingen, Deutschland*, [2]*BG Klinik Tübingen, Tübingen, Deutschland*, [3]*BG Unfallklinik Murnau, Murnau am Staffelsee, Deutschland*

**Background:**
The number of acetabular and pelvic fractures is increasing immensely. Pelvic ring fractures are the sixth most common fractures in adults [1], while most pelvic fractures occur in the elderly [2, 3]. Surgical-approach-related complications are reported with rates up to 31% [4]. Minimal invasive surgery techniques are sought [5, 6]. Augmented Reality (AR) has the capability to make invisible objects visible [7]. Aim of this study was to develop an AR-based, radiation-free navigation system for pelvic fractures.

**Materials and methods:**
The AR-tool was developed using a Microsoft® HoloLens 2 [8]. Unity® game engine (Version 2021.3.4f1 LTS) was used for programming [9]. Pelvic models by Sawbones® functioned as basis. A three-dimensional model was acquired utilizing CT-scans of the Sawbones model. Segmentation was done using Slicer3D by Slicer Corporation [10, 11]. The AR tool was deployed to the HoloLens using Unity® and Microsoft® Visual Studio 2022. The symphysis and both anterior superior iliac spines were defined as matching points. Vuforia Engine (Version 10.15) was used for three-dimensional model tracking [12]. 10 pelvic models from Sawbones® were used for testing. 20 S1-Screws and 20 S2-Screws were placed using only AR-navigation. The real pelvis was not visible for the proband. Placement of screws was controlled using CT.

**Results:**
The chosen matching points were marked by the surgeon and thus the virtual pelvis gets resized and matched automatically. The matching process took 3 min 28 s in average. A preoperatively chosen drill channel was shown to the surgeon. The surgical drill is positioned correctly by the surgeon using three-dimensional model tracking. Instant visual feedback about the direction of drilling is provided.
18 out of 20 S1-screws and 3 out of 20 S2-screws were placed correctly. The mean operation duration was 7 minutes for S1-screws and 5 minutes for S2-screws. The operation included machine drilling and screwing by hand. A reduction of the operation duration throughout the operations can be seen.

**Conclusion:**
Trajectories can be defined and visualized by the AR tool. A registration scan is not necessary with our matching idea. A proper radiation-free matching is possible. The drill channel can be visualized. Our tests show that a proper drilling is possible, especially for S1-screws aiming for a slightly bigger drilling channel than S2-screws. The handling is intuitive, and a learning process can be observed. Subsequent tests will be done on human bodies.

1. Rupp, M., et al., *The Incidence of Fractures Among the Adult Population of Germany–an Analysis From 2009 through 2019.* Dtsch Arztebl Int, 2021. **118**(40): p. 665-669.
2. Melhem, E., et al., *Epidemiology of pelvic and acetabular fractures in France.* Orthopaedics & Traumatology: Surgery & Research, 2020. **106**(5): p. 831-839.
3. Rinne, P.P., et al., *The incidence and trauma mechanisms of acetabular fractures: A nationwide study in Finland between 1997 and 2014.* Injury, 2017. **48**(10): p. 2157-2161.
4. Elmadağ, M., et al., *The Stoppa approach versus the ilioinguinal approach for anterior acetabular fractures: a case control study assessing blood loss complications and function outcomes.* Orthopaedics & Traumatology: Surgery & Research, 2014. **100**(6): p. 675-680.

Abstracts – DCK 2024 – 141st Congress of the German Society of Surgery – Leipzig, April 24–26 • DOI 10.1515/iss-2024-2006
Innov Surg Sci 2024; 9 (Special Suppl 1): s253–s279

5. Märdian, S., et al., *[Imaging examination procedures, navigation and minimally invasive procedures in acetabular surgery].* Unfallchirurgie (Heidelb), 2023. **126**(2): p. 89-99.
6. Stöckle, U., K. Schaser, and B. König, *Image guidance in pelvic and acetabular surgery—Expectations, success and limitations.* Injury, 2007. **38**(4): p. 450-462.
7. Carmigniani, J., et al., *Augmented reality technologies, systems and applications.* Multimedia Tools and Applications, 2011. **51**(1): p. 341-377.
8. Microsoft. *HoloLens 2*. 2023; Available from: https://www.microsoft.com/de-de/hololens/hardware.
9. Technologies, U. 2021 [cited 2021; Available from: https://unity.com.
10. Fedorov, A., et al., *3D Slicer as an image computing platform for the Quantitative Imaging Network.* Magnetic resonance imaging, 2012. **30**(9): p. 1323-1341.
11. Slicer. *3D Slicer*. 2020; Available from: https://www.slicer.org.
12. Inc., P. *Getting Started with Vuforia Engine in Unity*. 2021; Available from:

### Real-time surgery augmentation through vibro-acoustic sensing to enhance situational awareness during laparoscopy

ID: 317

Moritz Spiller[1], Thomas Sühn[1 2], Nazila Esmaeili[1 3], Axel Boese[4], Jana Adams[5], Rabi Raj Datta[5], Alfredo Illanes[1]

[1]*SURAG Medical GmbH, Leipzig, Deutschland*, [2]*Otto-von-Guericke University Magdeburg, Department of Orthopaedic Surgery, Magdeburg, Deutschland*, [3]*Justus Liebig University of Giessen, Department of Otorhinolaryngology, Head and Neck Surgery, Giessen, Deutschland*, [4]*Otto-von-Guericke University, INKA—Innovation Laboratory for Image Guided Therapy, Magdeburg, Deutschland*, [5]*Universitätsklinikum Köln, Klinik und Poliklinik für Allgemein-, Viszeral-, Tumor- und Transplantationschirurgie, Köln, Deutschland*

**Background:**

In conventional open surgery, surgeons rely on their senses (visual, tactile, auditory) to assess organs and tissue. In minimally invasive surgery, these senses are limited. Endoscopic video is the primary source of information, occasionally supplemented with depth data for a 3D view. To overcome this limitation, tactile information measurement devices have been proposed [2], but they pose challenges and cost concerns.

Surgical Audio Guidance (SURAG) is a novel concept that exploits vibro-acoustic (VA) waves generated by instrument tips interacting with tissue [3]. It doesn't require embedded sensors and offers haptic and navigational information. SURAG has shown promise in puncture identification [4] and palpation assessment [5, 6].

This study evaluates SURAG's potential to audify tool-tissue interactions in laparoscopy. Different materials and tasks on synthetic tissues with varying characteristics were tested. Preliminary results confirm VA signals can be transformed into audible ranges, and various tissues have distinct vibro-acoustic dynamics. User studies will follow to assess SURAG's benefits in surgical education and practice.

**Materials and methods:**

In the experimental setup (Figure 1), realistic laparoscopic data acquisition conditions were recreated. A foam-lined plastic box housed three trocars for instrument insertion: an endoscopic camera, grasper, and scissors (HICURA Metzenbaum Scissors, Olympus, Japan). Different tissue samples representing materials for both palpation (wood, felt, foam, ultrasound gel pad) and cutting (paper, carton, 1.5 mm diameter packaging string) tasks were placed inside.

Each participant completed 20 palpation and 20 cutting tasks on each tissue type, along with 20 cutting tasks in the air. A SURAG sensing module recorded vibro-acoustic (VA) signals produced during these interactions, providing real-time feedback. Simultaneously, endoscopic video data were collected for later analysis.

Post-analysis included annotating VA signals with task type, time, and material details, using Matlab R2022a. Qualitative analysis employed Continuous Wavelet Transformation (CWT) to study time domain, time-spectral energy dynamics (for cutting), and frequency component variations (for palpation). Quantitative analysis assessed CWT-derived instantaneous dominant frequency for each signal event segment, revealing distinct signal properties based on material and task.

Abstracts – DCK 2024 – 141st Congress of the German Society of Surgery – Leipzig, April 24–26 • DOI 10.1515/iss-2024-2006
Innov Surg Sci 2024; 9 (Special Suppl 1): s253–s279

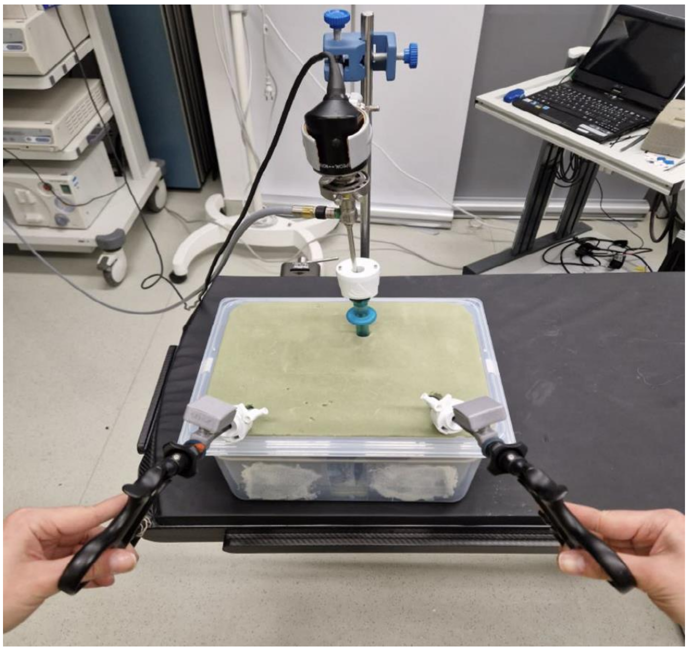

*Figure 1: The experimental setup for cutting and palpation tasks.*

**Results:**

Figure 2 displays acquired vibro-acoustic (VA) signals from five cutting tasks: scissors opening and closing in the air, cutting paper, carton, and packaging string. Distinct cutting events are visually identifiable in the VA signal, and tool-tissue interaction excitations are clearly distinguishable from the signal background, resulting in audible cutting sounds as feedback. Each cut material exhibits unique VA signal properties, enabling material differentiation. Carton cutting, for instance, produces different VA behavior due to its less homogeneous nature, resulting in a less smooth cut.

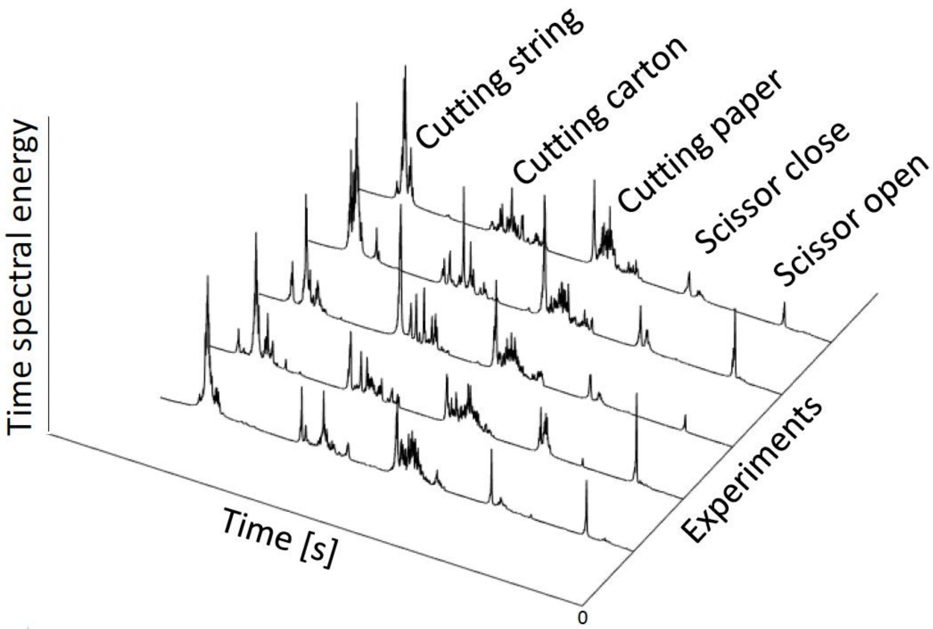

*Figure 2: VA signals acquired while cutting string, carton, and paper as well as while closing and opening the laparoscopic scissor. The observable signal characteristics allow the differentiation between the materials and the tasks.*

Figure 3 presents VA signals from palpating four materials: wood, ultrasound gel pad, foam, and felt. Notable differences in VA signals depending on the palpated material are evident. Even for softer materials like foam and gel pads, material differentiation is feasible in both the time and frequency domains. Primary tissue differences are observed in the 220 Hz to 920 Hz frequency band, where features can be extracted to allow AI-enabled tissue identification in laparoscopic surgery.

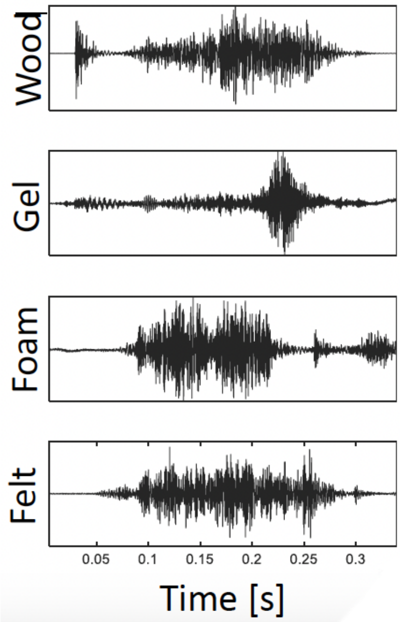

*Figure 3: VA signals acquired during palpation of wood, Ultrasound gel pad, foam and felt. It can be observed that the materials produce different signal characteristics, facilitating their differentiation.*

Figure 4 presents box plots that illustrate the variation in dominant frequency, also called the pitch, behavior during cutting (Figure 4a) and palpating (Figure 4b) tissues. The average dominant frequency feature differs among tissues for both cutting and palpation tasks. Specifically, when cutting the string, significantly higher frequencies are generated compared to other tissues. In the case of palpation, harder tissues such as wood exhibit higher frequency characteristics. The box plot for gel palpation demonstrates a larger dispersion, which can be attributed to the inherent instability of the interaction with the elastic Ultrasound pad. This instability can result in varied bumping behaviors that significantly influence the dominant frequency with each excitation. This finding is very important, since different dominant frequencies facilitate the ability of the surgeon to distinguish materials that are cut or palpated during surgery.

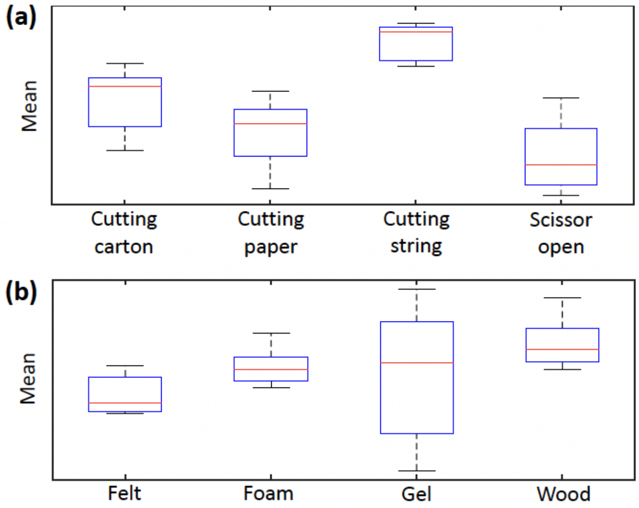

*Figure 4: Box plots for cutting (a) and palpation (b) events to assess the average of the dominant frequency.*

**Conclusion:**

This study proposed the use of vibro-acoustic sensing to augment laparoscopic surgery procedures. The analysis of vibro-acoustic signals obtained during cutting and palpation tasks on various tissue samples revealed that the signals resulting from tool-tissue interactions can be used to create acoustic feedback. Additionally, the results showed that the approach has the potential to allow surgeons to differentiate between tissue types and detect variations in tissue properties. These findings suggest that vibro-acoustic sensing could be integrated into the current clinical workflow to provide surgeons with an additional sensory modality complementary to the real-time video endoscope.

**References:**

[1] Boese A, Wex C, Croner R, Liehr UB, Wendler JJ, Weigt J, et al. Endoscopic imaging technology today. Diagnostics. 2022;12(5):1262.
[2] Bandari N, Dargahi J, Packirisamy M. Tactile sensors for minimally invasive surgery: A review of the state-of-the-art, applications, and perspectives. IEEE Access. 2019;8:7682-708.
[3] Illanes A, Sühn T, Esmaeili N, Maldonado I, Schaufler A, Chen CH, et al. Surgical Audio Guidance SurAG: extracting non-invasively meaningful guidance information during minimally invasive procedures. In: 2019 IEEE 19th international con-ference on bioinformatics and bioengineering (BIBE). IEEE; 2019. p. 567-70.
[4] Illanes A, Boese A, Maldonado I, Pashazadeh A, Schaufler A, Navab N, et al. Novel clinical device tracking and tissue event characterization using proximally placed audio signal acquisi-tion and processing. Scientific reports. 2018;8(1):12070.
[5] Chen CH, Sühn T, Kalmar M, Maldonado I, Wex C, Croner R, et al. Texture differentiation using audio signal analysis with robotic interventional instruments. Computers in biology and medicine. 2019;112:103370.
[6] Sühn T, Esmaeili N, Mattepu SY, Spiller M, Boese A, Urrutia R, et al. Vibro-Acoustic Sensing of Instrument Interactions as a Potential Source of Texture-Related Information in Robotic Palpation. Sensors. 2023;23(6):3141.

**Swarm Learning for Decentralized, Privacy-Preserving Collaboration in Surgical Data Science**
ID: 562

Fiona Kolbinger[1 2 3], Oliver Lester Saldanha[1], Sebastian Bodenstedt[4], Julia Barthel[2], Karolin Dehlke[2], Sophia Dietz[2], Guido Fitze[5], Martin Freitag[6], Thorsten Jacobi[7], Weam Kanjo[6], Sören Mees[8], Steffen Pistorius[2], Conrad Prudlo[5], Jurek Schultz[5], Astrid Seiberth[8], Daniel Ziehn[7], Jürgen Weitz[2], Stefanie Speidel[4], Jakob Kather[1], Marius Distler[2]

[1]*Technische Universität Dresden, Else Kröner Fresenius Zentrum für Digitale Gesundheit, Dresden, Deutschland*, [2]*Universitätsklinikum Dresden, Klinik für Viszeral-, Thorax- und Gefäßchirurgie, Dresden, Deutschland*, [3]*Purdue University, Weldon School of Biomedical Engineering, West Lafayette, IN, United States*, [4]*Nationales Centrum für Tumorerkrankungen, Abteilung für Translationale Chirurgische Onkologie, Dresden, Deutschland*, [5]*Universitätsklinikum Dresden, Klinik für Kinderchirurgie, Dresden, Deutschland*, [6]*Krankenhaus St. Joseph-Stift Dresden, Klinik für Allgemein-, Viszeral- und Endokrine Chirurgie, Dresden, Deutschland*, [7]*Diakonissenkrankenhaus Dresden, Klinik für Viszeralchirurgie und Proktologie, Dresden, Deutschland*, [8]*Städtisches Klinikum Dresden, Klinik für Allgemein- und Viszeralchirurgie, Dresden, Deutschland*

**Background:**
Limited availability and diversity of training data is the paramount obstacle in the development of clinically impactful Artificial Intelligence (AI) models in medicine. This problem is palpably present in surgical data science, where – compared to other medical fields such as radiology, histopathology, or dermatology – very few open-access datasets exist for model training and validation. Existing open-access datasets of intraoperative surgical imaging data mostly originate from less complex and relatively safe surgical procedures such as cholecystectomy. However, particularly in complex and less frequently performed operations such as oncological surgical procedures, the amount and diversity of training data required for the development and validation of generalizable, and thus clinically meaningful AI systems, can only be achieved through collaboration between medical institutions. Yet, patient data exchange between medical institutions is associated with serious concerns about data privacy and data protection. In practice, this results in diverse legal and logistic obstacles. Swarm Learning is a novel decentralized deep learning method that allows for confidential and democratic collaboration between scientific partners without data centralization. We address the challenges of data scarcity and data privacy in surgical data science through the application of Swarm Learning to predict outcome parameters based on various categorical, numerical, and imaging data.

**Materials and methods:**
As a proof-of-concept, we implemented Swarm Learning for automated prediction of the laparoscopic stage of appendicitis and other clinical endpoints including the duration of in-patient treatment based on clinical data and full-length recordings of laparoscopic appendectomies from six German centers. Data collection and analysis are currently ongoing. Deep Learning models are trained locally at collaborating institutions (Swarm Nodes) using exclusively data from the respective institution and are combined using blockchain-based communication within the network of participating institutions (Swarm Network).

**Results:**
Common challenges encountered during system setup at the participating institutions included practical challenges (i.e., technical requirements for systematical surgical video recording, different hospital infrastructure, data format compatibility) as well as challenges in data analysis (i.e., frame sampling rates in surgical video data, potential selection bias).
Appendectomy data collection and analysis are currently ongoing and will be finalized in Spring 2024. We expect to be able to present results on the prediction of relevant clinical endpoints from multicentric surgical data from six centers in April 2024.

**Conclusion:**

In this work, we systematically apply Swarm Learning to medical (imaging) data in surgery for the first time. The findings from our proof-of-concept in appendectomy as a use case will subsequently be transferred to more complex and clinically impactful use cases including oncological surgery.

Abstracts – DCK 2024 – 141st Congress of the German Society of Surgery – Leipzig, April 24–26 • DOI 10.1515/iss-2024-2006
Innov Surg Sci 2024; 9 (Special Suppl 1): s253–s279

**Mixed Reality Navigation in Neurosurgery: A Novel Registration Approach Using a Laser Crosshair Simulator for Accuracy and Efficiency Enhancing**
ID: 877

Ziyu Qi[1 2], Jiashu Zhang[2], Haitao Jin[2 3 4], Christopher Nimsky[1 5], Xiaolei Chen[2], Xinghua Xu[2], Qun Wang[2], Zhichao Gan[2 3], Ruochu Xiong[2 6], Shiyu Zhang[2 3], Jingyue Wang[2 3], Minghang Liu[2 3], Miriam H. A. Bauer[1 5]

[1]*University of Marburg, Department of Neurosurgery, Marburg*, [2]*First Medical Center of Chinese PLA General Hospital, Department of Neurosurgery, Beijing*, [3]*Medical School of Chinese PLA General Hospital, Beijing*, [4]*Army Medical University, NCO School, Shijiazhuang, China*, [5]*Center for Mind, Brain and Behavior (CMBB), Marburg, Deutschland*, [6]*Kanazawa University, Department of Neurosurgery, Division of Medicine, Graduate School of Medical Sciences,, Kanazawa, Ishikawa, Japan*

**Background:**
Commercial navigation systems have substantially revolutionized neurosurgery, seamlessly integrating preoperative images with physical patients. Initial systems, such as pointer-based navigation, offered groundbreaking precision yet suffered from ergonomic issues, i.e., frequent interruptions during operations and the need to shift attention. The introduction of Augmented Reality (AR) heralded a major technological advancement, particularly in microscope-based navigation. However, its reliance on extensive hardware components rendered it a costly investment, which spurred interest in more accessible and user-friendly AR alternatives, including those based on projectors, smartphones, tablets, and head-mounted displays (HMD) . Among these, HMD-based AR, especially the optical see-through paradigm, stood out as the frontrunner, offering a deeply immersive environment combined with the convenience of hands-free operation.

Into this mix came Mixed Reality Navigation (MRN), a subset of MR, tailored specifically for surgery. While MR, exemplified by devices such as Microsoft's HoloLens, offered an immersive 3D interaction model, MRN was designed to bring that immersion directly into the neurosurgical field. It aimed to provide neurosurgeons with a comprehensive 3D neuroanatomical interactive model at a fraction of the cost and complexity of traditional systems. Nonetheless, the rapid adoption of MRN brought to light several inherent challenges. The holographic registration process could have been more user-dependent, leading to potential inaccuracies. Moreover, MRN systems exhibited spatial localization errors, which posed substantial risks in the precise world of neurosurgery. Issues like hologram stability, real-time updates to surgical changes, and system lag further exacerbated these challenges. While MRN represents a promising step forward, these concerns underscore the need for refining its technological foundations, ensuring the system seamlessly aligns with the rigorous demands of neurosurgical applications.

**Materials and methods:**
Here, a novel registration approach is presented, rooted in using a laser crosshair simulator. This innovative strategy aims to accurately determine a coordinate origin consistent with computed tomography (CT) or magnetic resonance imaging (MRI) data , replicating the scanner frame's pose (position and orientation) on the patient. The devised system streamlines the process of transformation computation, transitioning coordinates from the tracking domain to the designated image context. Subsequently, a complete practical workflow was designed for this registration approach, culminating in creating a Universal Windows Platform (UWP) software named "MR Platform" specifically tailored for HoloLens-2.

The objective of the crosshair simulator is to transfer scanning parameters across varying temporal and spatial domains, enabling the determination of the acquisition location of the reference images. It primarily comprises two coplanar laser emitters mounted on a rack and a computer-vision recognizable target pattern. These emitters can project two sets of laser crosshairs, which are orthogonal to each other, into the simulator. The deployment of the crosshair simulator begins after the fixation of the patient's head. The surgeon activates the laser emitters and adjusts the simulator's pose until the projected lasers perfectly align with the laser positioning lines previously marked on the patient's skin. Once aligned, the simulator's pose is locked to maintain its relative position to the head. Subsequently, the MR platform on HoloLens-2 utilizes Vuforia's proprietary feature detection algorithm and known image targets on the MR interface to realize real-time tracking of the crosshair simulator's position, enabling visualization of the Reference Image Coordinate System (RICS). The holograms are then imported into RICS through gesture commands, achieving initial MRN registration.

To evaluate the proposed system's feasibility and accuracy, preoperative cranial CT or MRI imaging data were collected from 19 patients (male / female: 12/7, 54.5 ± 18.5 years), who were scheduled for neurosurgical intervention. Before the reference scanning, 6-7 CT or MRI visible fiducial markers were attached to the scalp of all patients. Imaging data was postprocessed, encompassing segmentation, annotation, tractography, and 3D reconstruction. The final 3D models compatible with HoloLens-2 include Lesions, ventricles, fiducials, arteries, veins, fiber tracts and scalp quadrants for rapid visual verification. Meanwhile, a 1:1 scale head phantom replicating the skin surface was produced for each case. The surgeon utilized the crosshair simulator to register the holograms to the phantom head using the MRN system.

To quantitatively assess the system's accuracy, the Target Registration Error (TRE) was measured, defined as the displacement vector between a holographic representation of the fiducial marker in the virtual space and its corresponding physical marker in the physical world. Since they were not involved in the registration process, the attached fiducial markers were chosen as known measurement reference points. For each reference point, a surgeon with extensive AR experience meticulously placed the tip of the virtual probe on the perceived physical-world marker. The platform instantly reported the 3D coordinates of the probe tip in RICS to the user's panel. Subsequently, the distance from the centroid of the marker in virtual space to the perceived physical-world marker (i.e., the magnitude of the TRE vector) could be calculated using the following equation:

$$\|\mathrm{TRE}_i\|_2 = \sqrt{\left(P_{ix} - C_{ix}\right)^2 + \left(P_{iy} - C_{iy}\right)^2 + \left(P_{iz} - C_{iz}\right)^2}$$

In the equation, $\|\cdot\|_2$ denotes the Euclidean norm of the vector, $P_i$ represents the perceived physical point in RICS, and $C_i$ signifies the centroid of the virtual marker in the RICS. In the context, $x$, $y$, and $z$ represent the components on the three principal axes of the RICS.

Statistical analysis was conducted using a one-way Analysis of Variance (ANOVA) to compare the accuracy under different "surgical positions," i.e., supine, prone, and lateral, with the significance level set at $p<0.05$.

**Results:**
All 19 cases achieved MRN visualization, enabling an immersive observation and direct 3D understanding of a set of holograms. An extensive evaluation across 124 measuring coordinate pairs harvested from these patient models yielded a TRE of 3.0 ± 1.1 mm. When comparing different operative positions, no statistically significant variations in TRE were discerned (2.9 ± 1.1 vs. 3.1 ± 1.1 vs. 3.0 ± 1.3 mm, $p = 0.673$).

**Conclusion:**
The comprehensive insights derived from the pilot development and subsequent evaluations underscore that the proposed method proffers an encouraging amalgamation of efficiency, intuitiveness, and accuracy. Such advancements not only epitomize a meaningful progression in affordable, standalone, and user-centric MRN systems but also highlight its latent potential to refine accuracy and adaptability in neurosurgical interventions, potentially ushering in improved surgical outcomes.

**Implementation of a Transdisciplinary Digital Health Curriculum – A One-Year Experience of Knowledge Transfer and Networking in a Unique Digital Health Training Culture**
ID: 13

Juliane Kröplin[1], Leonie Maier[1], Jan-Hendrik Lenz[1 2], Bernd Romeike[2]

[1]*Universitätsmedizin Rostock, Klinik und Poliklinik für Mund-, Kiefer- und Gesichtschirurgie, Rostock, Deutschland*, [2]*Universitätsmedizin Rostock, Studiendekanat, Rostock, Deutschland*

**Background:**
Digital health has been taught at medical faculties for a few years. Overall, however, the teaching of digital competencies in medical education and training is still underrepresented. This study aims to analyse the objective acquisition of digital competencies through the implementation of a transdisciplinary digital health curriculum at a German university. Furthermore, the analysis of the acceptance of the transdisciplinary approach as well as the application of an alternative examination performance (term paper) is carried out.

**Materials and methods:**
The main subject areas management/digital leadership, digital didactics, digital communication and robotics/artificial intelligence were taught over a period of one semester. The analysis period is one year. Relevant contents of the curriculum were indicated by the participants on a Likert scale of (1 = very well taught to 5 = not taught at all) regarding the competencies already taught in advance during the study. The participants' digital competencies were examined with a pre-posttest consisting of 12 questions. The learning objectives were assigned to the courses of our curriculum. The curriculum was available to students from all faculties of the University.

**Results:**
In the first year after the introduction of the elective, students of human medicine (n=15), dentistry (n=3), and medical biotechnology (n=2) participated in the curriculum. N=13 participants were female (male: n=7). 61.1% of the participants from human medicine and dentistry were in the preclinical study stage (clinical: 38.9%). All of the aforementioned learning objectives were largely absent from all study sections (preclinical: mean:4.2; clinical mean: 4.4, p=0.024). The pre-posttest comparison revealed a significant increase in knowledge of 106% (p<0,001) among the participants.

**Conclusion:**
The transdisciplinary teaching of a Digital Health curriculum considers perspectives and skills from different disciplines. Our new curriculum facilitates an objective increase in knowledge regarding the complex challenges of the digital transformation of our healthcare system. Out of 16 student term papers arising from the course, robotics/AI attracted the most interest, accounting for nine of the submissions.

### Surgical Wound Teaching - Easy to Implement for Improved Skills in the Future

ID: 48

Pernilla Conrad[1], Regine Gläser[2], Katrin Hertrampf[3], Gudrun Karsten[4], Charlotte Hauser[1], Thomas Becker[1]

[1]*Universitätsklinikum Schleswig-Holstein, Klinik für Allgemein-, Viszeral-, Thorax-, Transplantations- und Kinderchirurgie, Kiel, Deutschland*, [2]*Universitätsklinikum Schleswig-Holstein, Quincke-Forschungszentrum, Dermatologische Forschung , Kiel*, [3]*Universitätsklinikum Schleswig-Holstein, Klinik für Mund-, Kiefer- und Gesichtschirurgie, Kiel*, [4]*Christian-Albrechts-Universität zu Kiel , Studiendekanat der Medizinischen Fakultät , Kiel*

**Background:**
Wound healing disorders and infections often have far-reaching consequences for patients and represent a financial burden for the healthcare system. Wound management is relevant to every specialty and is currently taught inadequately in most medical curricula. The goal of this teaching project was to design and pilot a course to teach knowledge and practical skills in surgical wounds.

**Materials and methods:**
In WS 2022/23, the subjective knowledge of the students in the 2nd clinical year and in the practical year was surveyed by means of a questionnaire (Likert scale: 1=do not agree, 5=agree completely). Based on this, an innovative practical course was designed, consisting of a theoretical e-learning and a 3-hour practical course part. Learning success was assessed by means of a pre-post survey with a control group.

**Results:**
The survey (n=169) revealed a significant knowledge deficit, especially in practical skills (mean value: 2.2 vs. 2.4). Wound teaching was rated as inadequate (1.4 vs. 1.4). The pilot course was conducted in SoSe 2023 with 15 volunteer 2nd clinical year students on “lifelike wound models” and patients. The recommendation rate was 100%. Compared to the control group, there was a significant increase in knowledge (practical skills: 3.9 vs. 2.0). Participants felt more confident in managing surgical wounds (3.5 vs. 2.4).

**Conclusion:**
Wound assessment and management are basic skills that every medical practitioner must possess. Currently, practical training is insufficient. This gap can be closed with the help of a practical course on "lifelike wound models" and patients. This course can be easily implemented at any facility and provides the base for an interdisciplinary and professional curriculum.

### Using Virtual Reality for training surgeons in acetabulum surgery

ID: 176

Vincent Schenk[1 2], Alexander Trulson[3], Leyla Tümen[2], Tina Histing[2], Steven Herath[2], Markus Küper[2]

[1]*Eberhard Karls Universität Tübingen, Tübingen, Deutschland*, [2]*BG Klinik Tübingen, Tübingen, Deutschland*, [3]*BG Unfallklinik Murnau, Murnau am Staffelsee, Deutschland*

**Background:**
Fractures of the acetabulum are increasing continuously. From 2009 to 2019 an increase of 58% could be recorded [1]. Acetabular fractures mostly get classified according to Judet and LeTournel. The classification by the French surgeons contain 10 types of fracture patterns [2]. For young surgeons and medical students, the LeTournel classification presents a challenge. Using three-dimensional CT-scans can improve the correctness of classification compared to plain radiographs in unexperienced surgeons by up to 54% [3]. Virtual Reality (VR) describes an only artificial experience. The user moves and interacts with the virtual surrounding [4]. VR can be used for training young surgeons. It is used throughout all surgical fields [5].

**Materials and methods:**
An HTC® VIVE Pro was used for implementation. The VIVE Pro uses a head-mounted-display. The system can be operated by two hand controllers [6]. An Alienware m15 R4 functioned as VR-ready computer. Unity® (Version 2021.3.4f1 LTS) was used for programming. Unity is an open-source game engine. It is compatible with VR-programming and the HTC VIVE Pro [7]. SteamVR functioned as interface between the VIVE Pro and Unity® [8]. Segmentation was done utilizing Slicer3D [9, 10]. 11 pelvic models from real patients of our institution were integrated. Correct classification and drilling channels for each pelvis were chosen by experienced pelvic surgeons.

**Results:**
A VR-training system could be developed. The tool guides the user through 11 different levels. Every level containing a different pelvic model. Each pelvis shows a different fracture pattern. The user can move around freely through a virtual operating room. The pelvis can be grasped and turned in every degree of freedom. Every level guides the player through a certain process. The correct classification of the pelvis must be chosen by multiple choice. After choosing the correct classification according to LeTournel, the user is presented with a suitable osteosynthetic therapy. By grasping a virtual drill machine, the user can position ostheosynthetic screws into the pelvis. The positioning can be checked and corrected if necessary. An overview of the performance will be shown after passing all levels.

**Conclusion:**
It could be shown that a trainings simulator for young residents and medical students can be developed using only commercially available technology. The system is interactive and intuitive to use. An evaluation of the learning efficiency of the tool will be done in the future. The learning process will be evaluated on medical students. Correct classification of acetabular fractures in VR will be compared to 3D-CT-scans and regular plain radiographs.

1. Rupp, M., et al., *The Incidence of Fractures Among the Adult Population of Germany–an Analysis From 2009 through 2019.* Dtsch Arztebl Int, 2021. **118**(40): p. 665-669.
2. Judet, R., J. Judet, and E. Letournel, *FRACTURES OF THE ACETABULUM: CLASSIFICATION AND SURGICAL APPROACHES FOR OPEN REDUCTION. PRELIMINARY REPORT.* J Bone Joint Surg Am, 1964. **46**: p. 1615-46.
3. Hüfner, T., et al., *Classification of acetabular fractures. A systematic analysis of the relevance of computed tomography.* Der Unfallchirurg, 1999. **102**(2): p. 124-131.
4. Vince, J., *Introduction to virtual reality*. 2004: Springer Science & Business Media.

5. Li, L., et al., *Application of virtual reality technology in clinical medicine.* Am J Transl Res, 2017. **9**(9): p. 3867-3880.
6. HTC. *HTC Vive Pro Full Kit*. 2023 [cited 2023 14.01.]; Available from: https://www.vive.com/de/product/vive-pro-full-kit/).
7. Sinicki, A. *What is Unity? Everything you need to know*. 2021 [cited 2023; Available from: https://www.androidauthority.com/what-is-unity-1131558/.
8. Malventano, A. *STEAMVR HTC VIVE IN-DEPTH – LIGHTHOUSE TRACKING SYSTEM DISSECTED AND EXPLORED*. 2016 [cited 2023 16.01.]; Available from: https://pcper.com/2016/04/steamvr-htc-vive-in-depth-lighthouse-tracking-system-dissected-and-explored/2/#ftoc-heading-3.
9. Fedorov, A., et al., *3D Slicer as an image computing platform for the Quantitative Imaging Network.* Magnetic resonance imaging, 2012. **30**(9): p. 1323-1341.
10. Slicer. *3D Slicer*. 2020; Available from: https://www.slicer.org.

Abstracts – DCK 2024 – 141st Congress of the German Society of Surgery – Leipzig, April 24–26 • DOI 10.1515/iss-2024-2006
Innov Surg Sci 2024; 9 (Special Suppl 1): s253–s279

**Evaluation of Holomedicine® Masterclass for advanced medical training**
ID: 819

Daniela Salzmann[1], Marc-Angelo Bisotti[1], Verena Uslar[2], Navid Tabriz[2], Sirko Pelzl[1], Dirk Weyhe[2]

[1]*apoQlar GmbH, Hamburg, Deutschland*, [2]*Carl von Ossietzky Universität Oldenburg. Department für Humanmedizin, Universitätsklinik für Viszeralchirurgie, Pius-Hospital, Oldenburg, Deutschland*

**Background:**
New technologies like extended reality (XR) are currently finding many new applications in medicine and education. The new concept of virtual masterclasses, the Holomedicine® Masterclass, brings a wealth of new possibilities and can incorporate for example 3D virtual visualization and interaction, live streaming, interactive discussions, and even virtual reality simulations. The authors developed and designed the new Holomedicine® Masterclass training for experienced senior surgeons interested in using patient-specific 3D models with extended Reality (XR) technology for advanced visceral surgery. Since no virtual masterclass with such new technologies has been established before, the aim of this study is to evaluate the new concept and technology acceptance for the Holomedicine® Masterclass in the use of advanced medical training.

**Materials and methods:**
We will invite all participants, who attend the already scheduled Holomedicine® Masterclasses, to evaluate this new format upon completion. The course covers the latest advances in Holomedicine and provides practical training for preoperative planning, intraoperative guidance, and postoperative education. Participants will learn how to incorporate 3D models into surgical practice to improve patient outcomes. The evaluation consists of semi-structured qualitative interviews as well as an online survey. The items address the topics: potential usefulness, advantages / disadvantages, benefits, usability, user satisfaction and technology acceptance of the XR virtual Masterclass for advanced surgical training as well as sociodemographic, professional and technology experience of the attendees. The qualitative interviews will be recorded and transcribed. Finally, the qualitative data will be evaluated and summarized based on a qualitative content analysis within the framework of a category system. Furthermore, user satisfaction and technology acceptance will be analyzed by statistical descriptive data analysis.

**Results:**
The results of the ongoing evaluation will be presented. There will probably be 15 attendees participating in the evaluation. The first results from the pretest of the evaluation showed that the surgeons directly wanted to use it, and saw the high potential of the new training format due to the good usability, easy interaction with medical data, and possibility of direct communication with other attendees. One surgeon commented ‘Absolute future technology’. Based on the initial assessment the technology acceptance and user satisfaction seems to be high. A limitation was seen in the head-mounted headset because, which might make it too for heavy during prolonged use.

**Conclusion:**
**The** HoloMedicine® masterclass break down geographical barriers, are more time- and cost-effective, and provide a safe practice environment. However, it’s essential to acknowledge that Holomedicine® Masterclasses should complement, rather than replace, traditional hands-on training and mentorship. Surgeons must still gain practical experience. Additionally, at this early stage of implementation, continuous monitoring and evaluation are mandatory to ensure the quality, effectiveness, technology acceptance, and knowledge retention of Holomedicine® Masterclasses. Overall, Holomedicine® masterclasses in liver surgery hold immense potential to improve surgical education, expand access to expertise, and enhance patient care and patient safety. They are a valuable tool for both aspiring and experienced surgeons seeking to refine their skills and stay current with advancements in the field.

Abstracts – DCK 2024 – 141st Congress of the German Society of Surgery – Leipzig, April 24–26 • DOI 10.1515/iss-2024-2006
Innov Surg Sci 2024; 9 (Special Suppl 1): s253–s279

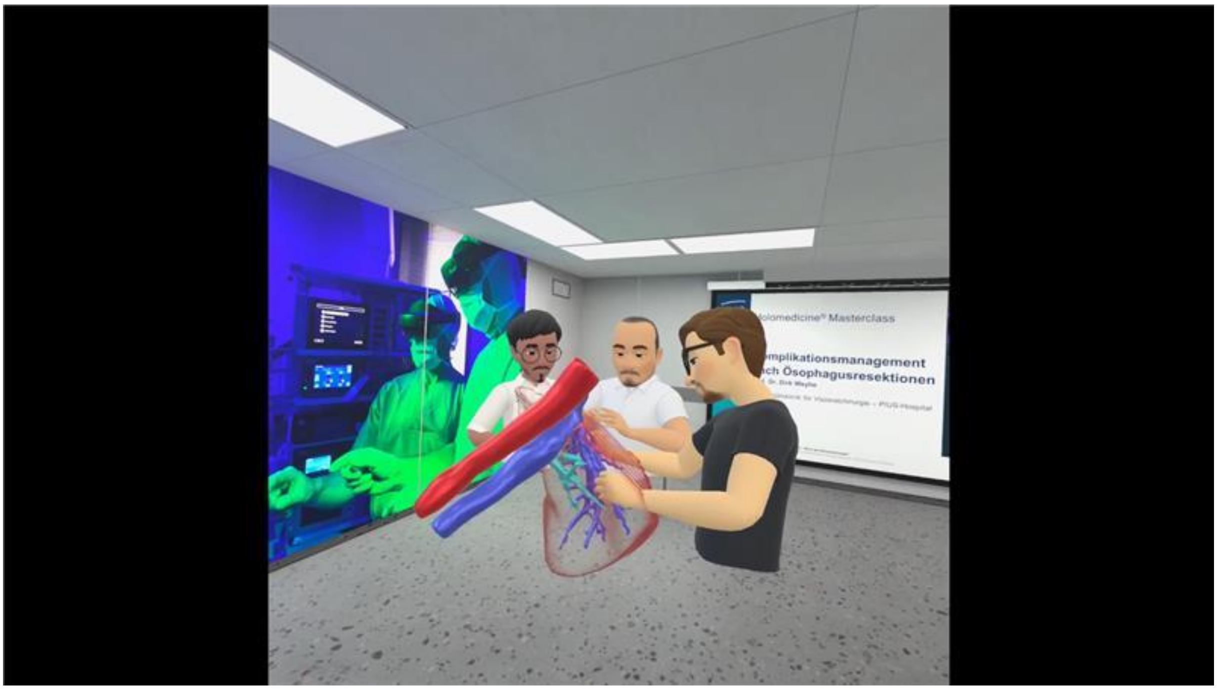

Picture 1: HoloMedicine® Masterclass session in liver surgery

**Quality of Outcome in Oral Cancer Patients - First Results of a Perioperative Evaluation of Patient-Specific Influencing Factors with Special Regard to Mental and Physical Strength**
ID: 31

Juliane Kröplin[1], Jil-Charlot Reppenhagen[1], Anke Hirsemann[2], Jan Liese[1], Bernhard Frerich[1]

[1]*Universitätsmedizin Rostock, Klinik und Poliklinik für Mund-, Kiefer- und Gesichtschirurgie, Rostock, Deutschland*, [2]*Universitätsmedizin Rostock, Onkologisches Zentrum, Rostock, Deutschland*

**Background:**
Quality assurance strategies for head and neck surgery can improve patient outcomes. The aim of the present study is the perioperative analysis of patient-specific factors influencing the quality of outcome in head and neck tumour surgery under university centre conditions.

**Materials and methods:**
Patients with oral squamosa cell carcinomas and indication for surgical resection were included in a prospective study. General and disease-related data as well as parameters of patient-specific resilience (RS-11 questionnaire) were collected. The general quality of life was analysed in terms of importance and satisfaction using the 9 items family/friends, sports activities/physiotherapy, participation in culture, professional perspectives, sexuality, enjoyable food, external appearance, social recognition and independence on a Likert scale (1 = very important/satisfied to 5 = not important/satisfied at all). The data collection took place pre- and postoperatively (mean: 10th postop day) during the inpatient stay.

**Results:**
N=17 patients with squamous cell carcinoma of the oral cavity were analysed (male: n=15, female n=3). The rate of R0 resections was 100%. The average length of stay was 21 days. N=7 of all patients was tracheotomised postop and n=4 patients at the time of discharge (preop: n=0). N=0 patients confirmed psycho(onco)logical support preop (postop: n=12). Sports activities/physiotherapy was provided to n=0 patients preop (postop: n=12). The mean postop pain perception was 3/10. The RS score decreased significantly postoperatively (mean: preop 5.3/7, postop: 4.1; $p<0.001$). A significant loss of satisfaction was seen in the areas of enjoyable food (mean: preop 2.1, postop 3.7; $p=0.002$), social recognition (mean: preop 2.4, postop 3; $p=0.029$), external appearance (mean: preop 2.2, postop 3.1; $p=0.011$) and independence (mean: preop 1.9, postop 3.3; $p=0.003$).

**Conclusion:**
Supportive therapy measures to promote the physical and mental fitness of oral cancer patients are part of inpatient care at our clinic. However, there is no structured monitoring of these therapies that can be measured and compared. Structured and measurable programmes to increase mental and physical fitness in both prehospital and inpatient care could lead to an improvement in the quality of outcome - such as higher resilience, lower perception of pain, earlier decannulation, and a better general quality of life. This might also improve the comparability of treatment strategies and postoperative courses.

Abstracts – DCK 2024 – 141st Congress of the German Society of Surgery – Leipzig, April 24–26 • DOI 10.1515/iss-2024-2006
Innov Surg Sci 2024; 9 (Special Suppl 1): s253–s279

**Clinically enlarged Lateral lymph node After Chemo-Radiation in rectal Cancer Patients: Surgery Versus Systemic Treatment - Systematic review and Meta-analysis update 2023.**
ID: 824

Ahmed Abdelsamad[1 2]

[1]*Ev. Krankenhaus Lippstadt, Lippstadt, Deutschland*, [2]*Universität Witten/Herdecke, Witten, Deutschland*

**Background:**
The lateral lymph nodes play a crucial role in the staging and treatment of rectal cancer. These lymph nodes are located along the sides of the mesorectum and are rarely involved in lymphatic drainage from this area. If the lateral lymph nodes are involved, this can impact the prognosis and treatment options. Understanding the significance of lateral lymph node involvement is essential for healthcare professionals in order to provide an appropriate and effective care for individuals with rectal cancer.
This meta-analysis aims to compare the efficacy of surgery versus systemic treatment in patients with clinically enlarged lateral lymph node after neoadjuvant chemo-radiation in rectal cancer. The study will include an update of systematic review of published literature on the topic and a meta-analysis of the data. The primary outcome measures will be overall survival, disease-free survival, and local recurrence. The secondary outcome measures will be complications, quality of life, and patient satisfaction.

**Materials and methods:**
This SRMA is registered with PROSPERO 2023 as ID: CRD42023457805 and available from: https://www.crd.york.ac.uk/prospero/display_record.php?ID=CRD42023457805. We applied our comprehensive terms in PubMed/Medline, Scopus, Cochrane library, and EMBASE and EBSCO central databases from January 2000 to September 2023. Retrieved references were gathered using EndNote in which we omitted the duplicates and exported the record for screening. Data regarding characteristics, short term- and long term outcomes, and baseline information of the enrolled population were extracted. Peer-reviewed scholarly research articles, case reports, case series, retrospective studies, clinical trials, and randomized controlled trials published in English and German languages, will only be included.
The study will include patients with clinically enlarged lateral lymph nodes after neoadjuvant chemo-radiation in rectal cancer who have undergone either surgery or systemic treatment. The quality of the studies will be assessed using the Cochrane risk of bias tool.

**Results:**
The results of this meta-analysis will provide valuable information on the efficacy of additional LLND with TME versus systemic treatment in patients with clinically enlarged lateral lymph node after neoadjuvant chemo-radiation in rectal cancer. This information can be used to guide clinical decision-making and improve patient outcomes.

**Conclusion:**
After conducting a meta-analysis on the management of lateral lymph nodes in rectal cancer patients, it can be concluded that addressing lateral lymph node involvement significantly improves patient outcomes. However, it is important to note that individual patient characteristics and tumor characteristics should be taken into consideration when determining the most appropriate treatment strategy. Further research and clinical trials are needed to refine treatment guidelines and optimize outcomes for patients with lateral lymph node involvement in rectal cancer.

**Pelvic exenteration for recurrent vulvar and endometrial cancer: a 15-year monocentric retrospective study**
ID: 916

Sabrina Claßen-von Spee[1], Nando Fix[1], Saher Baransi[1], Friederike Rawert[1], Veronica Luengas-Würzinger[1], Peter Mallmann[2], Björn Lampe[1]

[1]*Florence-Nightingale-Krankenhaus, Düsseldorf, Deutschland*, [2]*Uniklinik Köln, Köln, Deutschland*

**Background:**
Treatment options for recurrent vulvar and endometrial cancer are limited due to the presence of multifocal disease or applied radiotherapy. The diverse patient population impairs standardized procedures and asks for individualized treatment options. If patients already received or failed to respond to radio- or chemotherapy, secondary surgical procedures such as pelvic exenteration (PE) might be the only curative option. The heterogeneity of the published data is a big challenge for an interpretation of long-term survival after PE. Data for systemic therapy is rare and without clear approvals. Even in palliative intention in patients with distant metastasis or osseous/nerval infiltration PE can improve quality of life. The aim of this study was to analyze the outcomes of patients who underwent PE during the treatment of recurrent endometrial and vulvar cancer aiming to identify positive prognostic factors. Therefore, data from our oncological center was analyzed and correlated with the current literature.

**Materials and methods:**
A cohort of 32 patients received pelvic exenteration for recurrent vulvar (n=17) and endometrial adenocarcinoma (n=15) at the Florence-Nightingale-Hospital (Düsseldorf, Germany) during 2007 and 2022. Data regarding patient characteristics, indication for surgery (curative vs. palliative), complete cytoreduction, presence of lymphatic or distant metastases, tumor grading, blood- or lymphatic vessel invasion, p16, p53- and L1CAM-expression were collected and statistically evaluated. Univariate Cox regression was performed to identify predictive factors for long-time survival.

**Results:**
For vulvar cancer the mean age was 64.9 years (range 41 – 81 years) and 65.9 years (range 54 – 76 years) for endometrial cancer.
Mean survival after PE for vulvar cancer was 55.7 months, with the longest survival reaching up to 164 months. Patients with endometrial cancer showed a mean survival of 22.7 months, the longest time of survival was 69 months.
In case of vulvar cancer the overall survival was significantly affected by achievement of complete cytoreduction (p= 0,02). Strong indicators for overall survival were the presence of distant metastases (p= 0,06) and treatment intention (curative vs. palliative; p= 0,06). Lymphatic metastases were an unfavourable factor for patient survival (p= 0,11). Age, tumor grading, lymphatic- and blood-vessel invasion, as well as p53- and p16-expression showed no effect.
For endometrial cancer the overall survival was significantly longer for patients with a curative treatment intention (p = 0,015) and for patients with a well or moderately differentiated (G1 or G2) adenocarcinoma (p = 0,014). Achievement of complete cytoreduction seemed favourable. No effects on patient survival were measured with regards to p53- and L1CAM-expression or lymphatic- and blood-vessel invasion.

**Conclusion:**
Pelvic exenteration forms a possible treatment option for selected patients with recurrent vulvar or endometrial cancer. This surgical approach might offer a substantial prognostic improvement for patients with highly limited treatment options.

**Association of quantitative radiomic shape features with functional outcome after surgery for primary sporadic dorsal spinal meningiomas**
ID: 309

Martin Vychopen[1], Arlt Felix[1], Florian Wilhelmy[1], Clemens Seidel[2], Alonso Barrantes-Feer[3], Erdem Güresir[1], Johannes Wach[1]

[1]*University of Leipzig, Department of Neurosurgery, Leipzig, Deutschland*, [2]*University of Leipzig Medical Center, Department of Radiation Oncology, Leipzig, Deutschland*, [3]*University of Leipzig, Paul Flechsig Institute of Neuropathology, Leipzig, Deutschland*

**Background:**
Spinal meningiomas (SM) account for 25-46% of all primary spinal tumors and show an excellent long-term disease control in case of complete resection. Therefore, the postoperative functional outcome is of high importance. [1,2] To date, reports on dorsally located SM are scarce. Moreover, the impact of radiomics shape features on the functional outcome after surgery for primary dorsal SMs has not been analyzed yet.

**Materials and methods:**
We retrospectively performed an analysis of shape-based radiomic features in 3D slicer software and quantified the tumor volume, surface area, sphericity, surface area to volume ratio and tumor canal ratio. [3] Subsequently, we evaluated the correlation between the radinomic parameters and the postoperative outcome according to Modified Japanese Orthopedic Association (mJOA) score.

**Results:**
Between 2010 and 2022, we identified 24 Females and 2 Males operated on dorsal SMs in our institutional database. The most common SM localization was thoracic spine ($n$ = 20), followed by cervical ($n$ = 4), and lumbar ($n$ = 2). The univariate analysis and the receiver operating characteristic (ROC) analysis showed a strong diagnostic performance of sphericity in the prediction of postoperative functional outcome based on mJOA score (AUC of 0.79, sphericity cut-of value 0.738; $p$ = 0.01). Subsequently, the patients were divided into two groups (mJOA improved vs. mJOA stable/worsened). Patients with improved mJOA score showed significantly higher sphericity (0.79 ± 0.1 vs. 0.70 ± 1.0; $p$ = 0.03). Finally, we divided the cohort based on sphericity (<0.738 and ≥0.738). The group with higher sphericity exhibited a significantly higher positive mJOA difference 3 months postoperatively (16.6 ± 1.4 vs. 14.8 ± 3.7; $p$ = 0.03).

**Conclusion:**
In our study investigating primary sporadic dorsal SMs, we demonstrated that a higher degree of sphericity may be a positive predictor of postoperative improvement, as indicated by the mJOA score.

1. Aboul-Enein HA, Khidr WM, Abdeen KM, Madawi AA. Surgical management of ventrally based lower cervical (subaxial) meningiomas through the lateral approach: Report on 16 cases. Clin Neurol Neurosurg. 2015; 139: 152–158 https://doi.org/10.1016/j.clineuro.2015.10.008.
2. Alafaci C, Grasso G, Granata F, Salpietro FM, Tomasello F. Ossified spinal meningiomas: Clinical and surgical features. Clin Neurol Neurosurg. 2016; 142: 93– 97 https://doi.org/10.1016/j.clineuro.2016.01.026.
3. Fedorov A, Beichel R, Kalpathy-Cramer J, Finet J, Fillion-Robin JC, Pujol S, Bauer C, Jennings D, Fennessy F, Sonka M, Buatti J, Aylward S, Miller JV, Pieper S, Kikinis R. 3D Slicer as an image computing platform for the Quantitative Imaging Network. *Magn Reson Imaging.* 2012 Nov;30(9):1323-41 https://doi.org/10.1016/j.mri.2012.05.001
